# Photoperiodic changes in adiposity increase sensitivity of female Siberian hamsters to systemic VGF derived peptide TLQP-21

**DOI:** 10.1371/journal.pone.0221517

**Published:** 2019-08-29

**Authors:** Carlo Lisci, Jo E. Lewis, Zoe C. T. R. Daniel, Tyler J. Stevenson, Chloe Monnier, Hayley J. Marshall, Maxine Fowler, Francis J. P. Ebling, Gian-Luca Ferri, Cristina Cocco, Preeti H. Jethwa

**Affiliations:** 1 NEF-Laboratory, Dept. of Biomedical Sciences, University of Cagliari, Cagliari, Italy; 2 School of Life Sciences, University of Nottingham Medical School, Nottingham, United Kingdom; 3 Institute of Metabolic Sciences and MRC-Metabolic Diseases Unit, University of Cambridge, Cambridge, United Kingdom; 4 School of Biosciences, University of Nottingham Sutton Bonington Campus, Nottingham, United Kingdom; 5 Institute for Biodiversity, Animal Health and Comparative Medicine, University of Glasgow, Glasgow, United Kingdom; University of Minnesota, UNITED STATES

## Abstract

TLQP-21, a peptide encoded by the highly conserved *vgf* gene, is expressed in neuroendocrine cells and has been the most prominent VGF-derived peptide studied in relation to control of energy balance. The recent discovery that TLQP-21 is the natural agonist for the complement 3a receptor 1 (C3aR1) has revived interest in this peptide as a potential drug target for obesity. We have investigated its function in Siberian hamsters (*Phodopus sungorus*), a rodent that displays natural seasonal changes in body weight and adiposity as an adaptation to survive winter. We have previously shown that intracerebroventricular administration of TLQP-21 reduced food intake and body weight in hamsters in their long-day fat state. The aim of our current study was to determine the systemic actions of TLQP-21 on food intake, energy expenditure and body weight, and to establish whether adiposity affected these responses. Peripheral infusion of TLQP-21 (1mg/kg/day for 7 days) in lean hamsters exposed to short photoperiods (SP) reduced cumulative food intake in the home cage (p<0.05), and intake when measured in metabolic cages (P<0.01). Energy expenditure was significantly increased (p<0.001) by TLQP-21 infusion, this was associated with a significant increase in uncoupling protein 1 mRNA in brown adipose tissue (BAT) (p<0.05), and body weight was significantly reduced (p<0.05). These effects of systemic TLQP-21 treatment were not observed in hamsters exposed to long photoperiod (LP) with a fat phenotype. C3aR1 mRNA and protein were abundantly expressed in the hypothalamus, brown and white adipose tissue in hamsters, but changes in expression cannot explain the differential response to TLQP-21 in lean and fat hamsters.

## Introduction

Siberian hamsters (*Phodopus sungorus*) undergo natural seasonal cycles of adiposity. They are hyperphagic and accumulate large fat reserves in the summer under long photoperiods (LP), but in response to short (winter) photoperiods (SP) they enter a catabolic state where they reduce their food intake, and catabolize intra-abdominal fat reserves, subsequently reducing body weight by a third [1, 2]. Recently tanycytes, which are glial cells lining the third ventricle and projecting to the surrounding hypothalamus, have been shown to be an important part of the mechanism that facilitates seasonal physiology and behaviour in Siberian hamsters through their role in the regulation of local thyroid hormone availability [3, 4]. We have shown that thyroid hormones, namely tri-iodothyronine, decrease the expression of *vgf* mRNA *in vitro* and *in vivo* in hamsters exposed to SP [5].

Hypothalamic *vgf* mRNA expression is altered by photoperiod in the Siberian hamster, with significantly lower expression in SP when compared to LP in the hypothalamus/arcuate nucleus (ARC). However, upregulation is apparent in a sub-division of the ARC, the dorsomedial posterior ARC (dmpARC). Switching from SP to LP results in rapid decreases in *vgf* mRNA expression in the dmpARC ahead of body weight increases [6]. We have shown that over-expression of *vgf* in the hypothalamus using a rAAV strategy increases energy expenditure and reduces body weight gain in hamsters in LP [7], consistent with our previous observations that infusion of TLQP-21 into the hypothalamus exerts catabolic actions [8].

Pro-VGF is cleaved into a number of peptides, of these TLQP-21 has been most studied in relation to energy metabolism [9]. *In vivo*, TLQP peptides are expressed in many endocrine locations, including pancreatic islets [10], gastric endocrine cells [11] and the reproductive tract [12, 13], as well as in the nervous system, including the noradrenergic innervation of adipose tissue [14] [15]. The abundance of peptides containing the TLQP sequence including TLQP-21 itself show distinct changes upon feeding / fasting, or glucose loading, and in mouse and human obesity [11, 15], while they remain unaffected in other conditions, in which different VGF-derived peptides show a distinct response [16].

The recent discovery that TLQP-21 is a natural agonist for the complement 3a receptor 1 (C3aR1) has stimulated interest in this peptide as a potential drug target for obesity [17]. Although we have previously shown that central administration of TLQP-21 decreases body weight in LP-exposed Siberian hamsters [8], the effect of systemic administration is unknown. Here we show that chronic subcutaneous administration of TLQP-21 increased energy expenditure and reduced body weight in lean SP-exposed hamsters, but had no effect in animals exposed to LP. Furthermore, we show that photoperiod (SP vs LP) altered the expression of C3aR1 in the hypothalamus and brown adipose tissue (BAT), but not in white adipose tissue depots (WAT).

## Methods

### Peptide

Synthetic TLQP-21 (TLQPPASSRRRHFHHALPPAR based on the *Rattus norvegicus* genome) was used in all experiments. We would like to thank Dr Perry Barrett, University of Aberdeen, who independently verified the sequence against the *Phodopus sungorus* genome (accession number PRJNA318271; [18]), and observed that the sequences are identical.

### Animals

All animal procedures were approved by the University of Nottingham Animal Welfare and Ethical Review Board and were carried out in accordance with the UK Animals (Scientific Procedures) Act 1986 (project licence PPL 40⁄3604). Female Siberian hamsters aged 3 months were singly housed and transferred to short photoperiod (SP: 8h light/16h dark, lights off at 11:00) under controlled temperature (21±1°C) and on a reverse photoperiod with *ad libitum* access to food (Teklad 2019, Harlan, UK) and water. Body weight, food intake and pelage were assessed every two weeks. Pelage colour was evaluated on a nominal scale ranging from 4 (dark summer fur) to 1 (white winter fur) as previously described [19]. Age-matched controls were maintained in long photoperiod (LP: 16h light/8h dark), and were housed individually one week prior to implantation of osmotic minipumps.

### Metabolic cages

Oxygen consumption (VO_2_) and carbon dioxide production (VCO_2_) were measured concurrently using a modified open-circuit calorimeter known as comprehensive laboratory animal monitoring system (CLAMS) as previously described [7, 8]. VO_2_ and VCO_2_ were used to calculate energy expenditure and the respiratory exchange ratio as previously described [7, 8, 20]. Measurements were taken at 9 minute intervals for 48h; the first 24h of data were considered to be an acclimatisation period, so data were only analysed for the second 24h.

### Chronic treatment of hamsters with TLQP-21

Siberian hamsters in SP or LP (n = 8 per treatment per photoperiod) received a subcutaneously implanted Alzet osmotic mini-pump (model 1007D, Charles River) releasing vehicle (saline) or rat-TLQP-21 (1mg/kg/day) for 7 days as previously described [21]. Briefly, mini-pumps were inserted below the skin on the flank of the Siberian hamster under 1.5% isoflurane anaesthesia. Hamsters were treated with analgesic (5 mg/kg s.c., maintained for 3 days with additional fluids, 0.5 ml/day, Rimadyl, Pfizer, Kent, UK) and the wound closed with Michel clips. Body weight and food intake were recorded daily, shortly before lights out. Three days post-surgery animals were transferred to metabolic cages for 48h. At the end of the study (7 days post-surgery) hamsters were euthanized via an intraperitoneal (i.p.) injection of sodium pentobarbitone (Euthatal, Rhone Merieux, Harlow, UK). Samples of the hypothalamus, interscapular BAT (BAT) and interscapular, intra-abdominal and peri-renal WAT (iWAT, aWAT and prWAT respectively) were collected, snap frozen on dry ice and stored at −80°C until analysed.

### Quantitative real time PCR analysis

Uncoupling protein 1 (UCP1) and C3aR1 mRNA were measured as previously described [8]. Briefly, total RNA was extracted from 20 mg of frozen wet BAT, iWAT and prWAT using TRIzol reagent (Invitrogen). Aliquots of RNA were assessed for purity and quantified via Nanodrop ND-100 (Thermo Fisher Scientific, Wilmington, USA). Reverse transcription was carried out using 500 ng of total RNA using the SuperScript III cDNA kit (Invitrogen). Taqman primers and probes sets were obtained from Applied Biosystems (Table 1) using the Siberian hamster sequence and assembly (Accession: PRJNA318271 ID: 318271). Real-time PCR was performed using PCR Universal Master Mix (Applied Biosystems) in a Micro-Amp 96-well plate using an ABI Prism 7000 Sequence Detection System (Applied Biosystems). Assays were performed in triplicate. The threshold (Ct) values for each triplicate were averaged and the quantification of expression of each gene relative to β-actin or anti36B4 determined using the standard curve method.

**Table 1:** Primers obtained from Applied Biosystems.

*Gene*	*Forward primer*	*Reverse primer*
***UCP-1***	CCGGCTTCAGATCCAAGGT	TCGGCAACCCTTCTGTTTTT
***β-actin***	CGTGCGTGACATCAAAGAGAA	AGCAGTGGCCATCTCTTGCT
***C3aR1***	TGCCTCTCCTTGCCCTTCT	GTTTGCACAGGAACAAGCCATA
***36B4***	TCCAGGCTTTGGGCATCA	TTATCAGCTGCACATCACTCAGAAT

### Protein determination of C3aR1 in tissue

C3aR1 protein levels were determined via Western blot as previously described [22, 23]. Briefly, proteins were extracted from the hypothalamus and intra-abdominal WAT (aWAT) by homogenisation in cold HEPES Lysis buffer (50mM HEPES, 10% glycerol, 1mM EDTA, 10mM sodium fluoride, 1mM Sodium othovanadate, 150mM sodium chloride, 1% Triton X-100; pH 7.5) followed by centrifugation (10,000 G, 20 min, 4°C). Supernatants containing 20–50μg of protein were mixed with an equal volume of loading buffer (Laemmli loading buffer). Consistent amounts of protein were loaded, and separated on 4–15% precast acrylamide gel (Criterion TGX, BioRad), hence transferred electrophoretically to nitrocellulose membranes. Ponceau staining was used to confirm the equal loading of protein. Membranes were probed with a 1:1000 dilution of rabbit polyclonal anti-C3aR1 (Abcam, Cambridge, MA) and mouse monoclonal anti-βactin (Abcam, Cambridge, MA) or rabbit monoclonal anti αtubulin (Cell Signalling Technology, UK) antibodies overnight at 4°C followed by incubation with secondary goat-anti rabbit and goat-anti mouse HRP-linked antibody (1:2000–5000) for 1 hour at room temperature. Bands were visualized either using LI-COR Odyssey 9120 Imaging System (LI-COR Biosciences UK, Cambridge, UK) or using ECL detection reagent (GE Healthcare Life Science, UK) followed by exposure to photographic film in a dark room. Densitometry of band intensity was conducted to determine protein expression levels.

### Statistical analysis

Descriptive statistics (mean±SEM) were generated using Prism software (v6.0, GraphPad, San Diego, CA, USA). After checking for normality of distribution and equality of variance, body weight, food intake and CLAMS data were analysed using a two-way ANOVA (effect of time (repeated measured) vs. effect of treatment) followed by a Bonferroni *post-hoc* tests when a significant main effect or interaction was detected. Metabolite, mRNA and Western blot were analysed by Student’s unpaired T-tests. No animals were excluded from the analysis. P<0.05 was considered to indicate statistically significant differences.

## Results

### Effect of photoperiod

**Fig 1:**
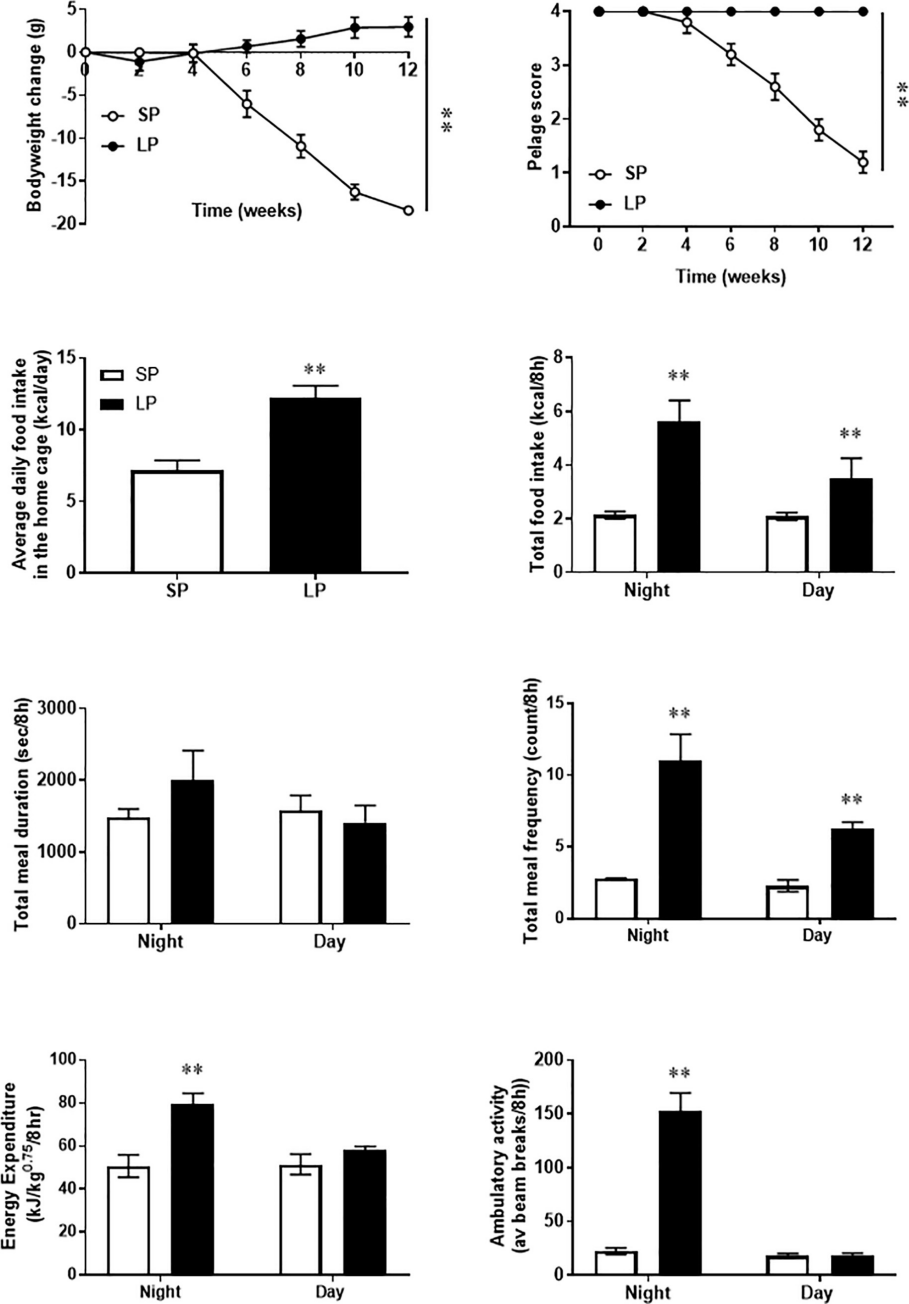
Exposing Siberian hamsters to short photoperiod (SP) reduces body weight, pelage and alters feeding behaviour while increases energy expenditure and activity. Female Siberian hamsters (n = 20/photoperiod) were maintained under long or short photoperiod for 12 weeks. Body weight (from day 0 of exposure; **A**), pelage (1 = winter, 4 = summer; **B**) and food intake (as daily average over 12 weeks; **C**) was determined over the 12 weeks in the home cage. Feeding behaviour [total intake (**D**), duration (**E**) and frequency (**F**) of intake], energy expenditure (**G**) and ambulatory activity (**H**) were determined in an automated animal monitoring system (CLAMS) after 12 weeks of exposure to short (SP) or long (LP) photoperiods, prior to any experimental manipulations. Metabolic cage data are expressed per 8 hours to normalise for different durations of light and dark phases. Open circles/white bars indicated SP exposure and closed circle/black bars indicated LP exposure. Values are expressed as mean ± SEM, **P<0.01 vs LP.

As expected, Siberian hamsters exposed to SP had a progressive loss of body weight that was significantly lower than that in those maintained in LP (P<0.001; Fig 1A). Mean weight loss at the start of the TLQP-21 treatment in SP was -18.5±0.5%. The pelage of hamsters in SP molted from agouti to white (P < 0.001; Fig 1B), and their average daily food intake was significantly reduced by 12 weeks of exposure to LP whether measured in the home cage (Fig 1C; P<0.01 or in the CLAMS (Fig 1D, P<0.01). The reduced daily food intake mainly reflected a reduction in the frequency of meals (eating bouts) (P<0.01), as the duration of meals was not significantly affected by photoperiod Fig 1E and 1F). Dark phase energy expenditure was reduced in hamsters exposed to SP (P<0.05, Fig 1G), this largely reflected significantly reduced ambulatory activity (P< 0.001; Fig 1H).

### Effect of chronic treatment with TLQP-21

**Fig 2:**
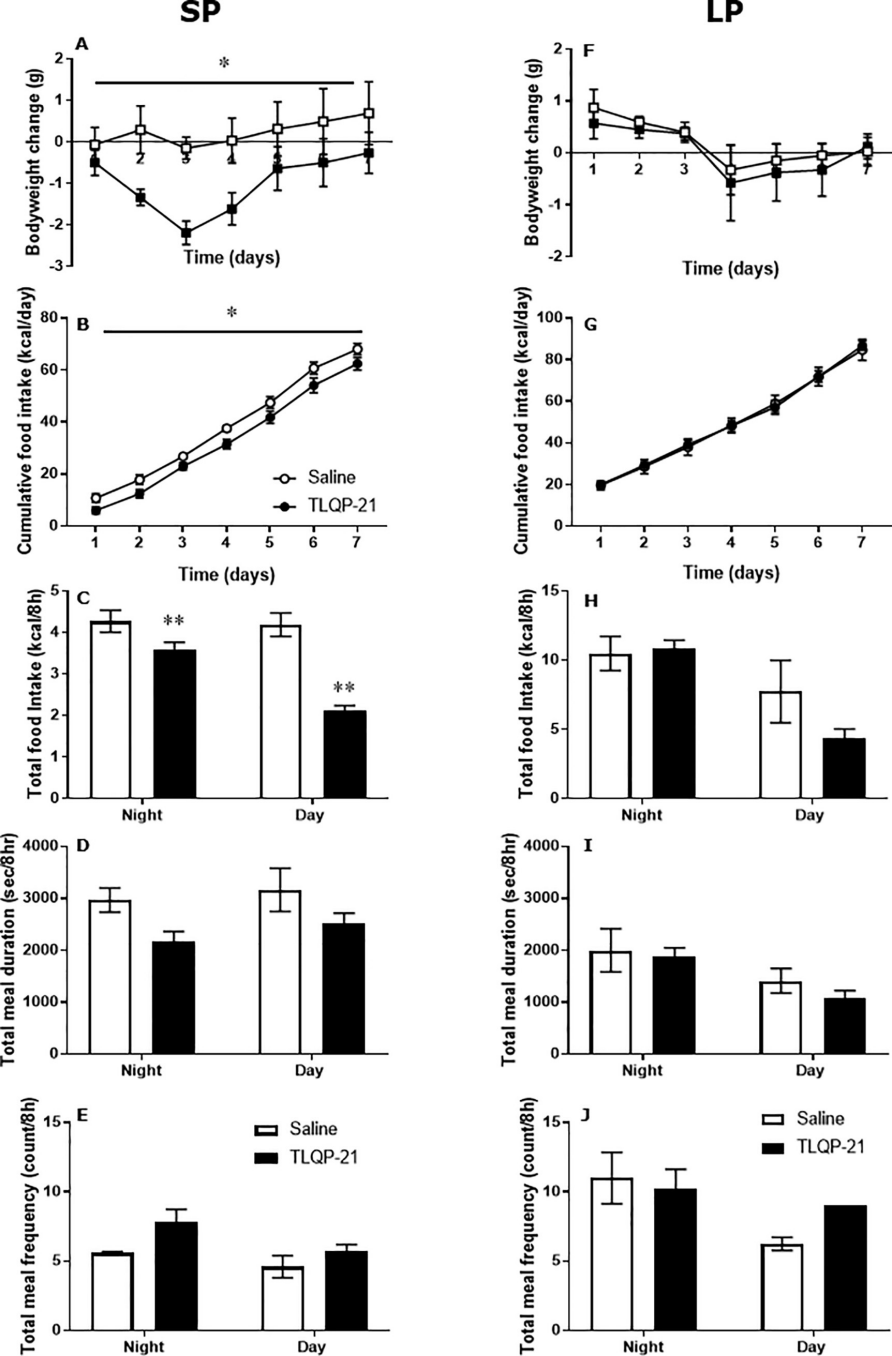
Chronic treatment with TLQP-21 induces further reductions in body weight and food intake in Siberian hamsters exposed to SP but not LP. Female Siberian hamsters (n = 8/treatment/photoperiod) exposed to 12 weeks of either SP and LP received chronic infusion of TLQP-21 (1mg/kg/day) or saline vehicle via an osmotic mini pump for seven days. Body weight change from pre-infusion weight (**A/F**) and cumulative food intake (**B/G**) were measured daily in the home cage. Feeding behaviour [total intake (**C/H**), meal duration (**D/I**) and meal frequency (**E/J**)] were measured over 24 hours in the automated animal monitoring system (CLAMS), beginning 3 days after the start of infusions. Metabolic cage data are expressed per 8 hours to normalise for different durations of light and dark phases. Open circles/white bars indicated vehicle treatments and closed circle/black bars indicated TLQP-21 treatments. Graphs **A-E** indicate SP exposed hamsters while **F-J** indicate LP exposed hamsters. Values are expressed as mean±SEM, *P<0.05; **P<0.01 vs vehicle control.

**Fig 3:**
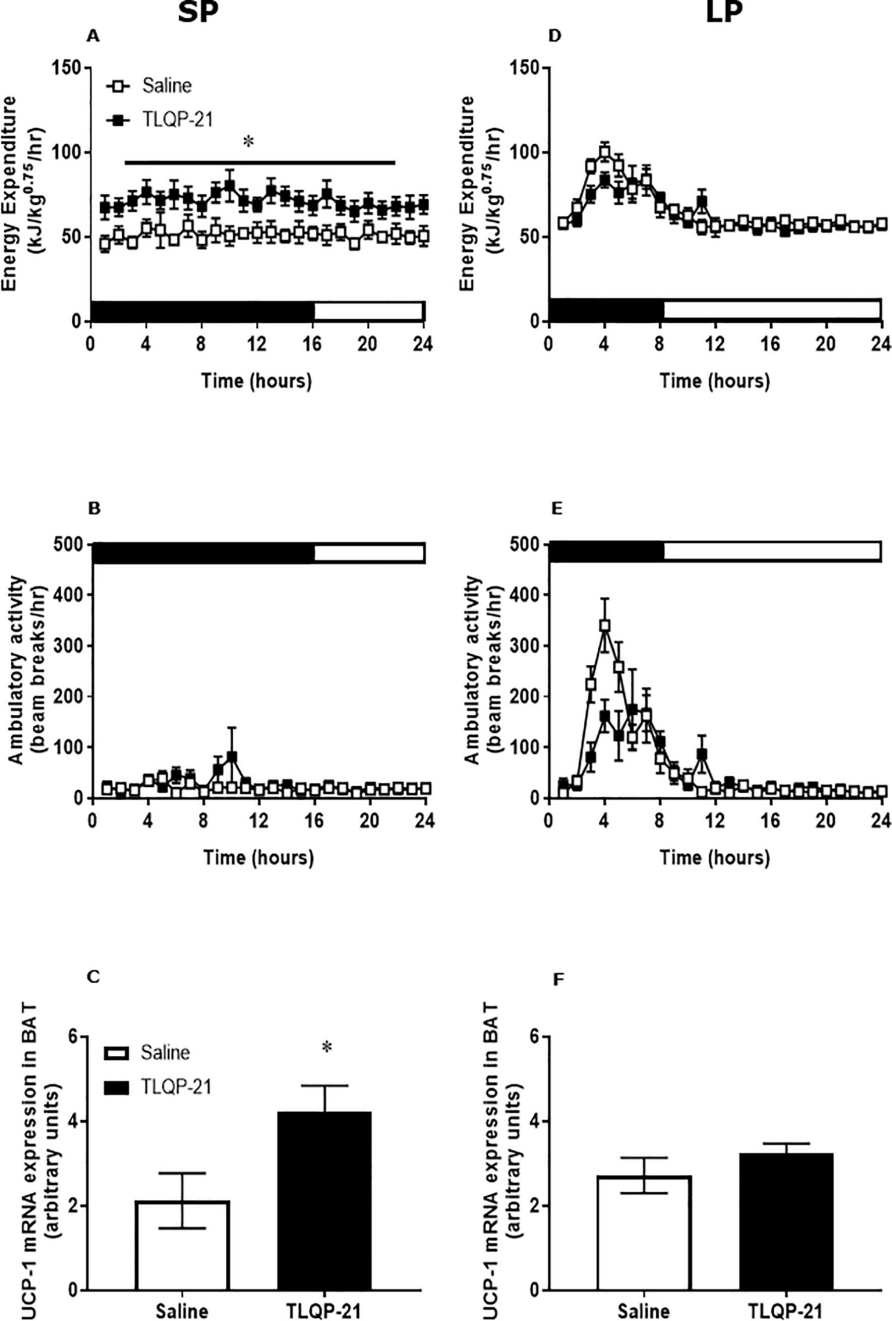
Chronic treatment with TLQP-21 increases energy expenditure in Siberian hamsters exposed to SP, but not to LP, associated with increase in UCP-1 expression in BAT. Siberian hamsters (n = 8/treatment/photoperiod) exposed to 12 weeks of SP and LP received chronic infusion of TLQP-21 (1mg/kg/day) or saline vehicle via osmotic mini pump for seven days. Twenty-four hour profiles of energy expenditure (**A**, **D**) and activity (**B**, **E**) were measured in metabolic cages beginning 3 days after the start of infusions. The different light-dark cycles on SP and LP are depicted by white and dark bar bars. **C, F** UCP-1 mRNA in BAT at the end of the studies (day 7) in SP and LP. Values are expressed as mean±SEM, *P<0.05 vs saline vehicle controls.

Systemic treatment of Siberian hamsters in SP with TLQP-21 via osmotic mini-pumps significantly reduced body weight (effect of treatment; F = 4.88, P<0.05; Fig 2A). This was associated with reduced cumulative food intake in their home cage (effect of treatment; F = 5.67, P<0.05; Fig 2B). This reduction in food intake was due to reduced meal intake (treatment vs, time interaction: F = 18.6, P<0.005; effect of treatment: F = 35.8, P<0.001; Fig 2C) during the dark phase. Interestingly, there was a trend towards both decreased meal duration (effect of treatment: p = 0.06; Fig 2D) and increased meal frequency (effect of treatment: p = 0.07, Fig 2E) in the hamsters treated with TLQP-21. In addition, a significant increase in energy expenditure was observed in response to treatment with TLQP-21 (effect of treatment: F = 5.41, P<0.001, Fig 3A), while ambulatory activity remained unchanged (Fig 3B). UCP1 mRNA in BAT was significantly increased following treatment with TLQP-21 (P< 0.05, Fig 3C).

Comparable effects of the TLQP-21 infusion were not found in fat Siberian hamsters in LP. Body weight (Fig 2F), cumulative food intake (Fig 2G), and feeding behaviour (meal duration, intake and frequency (Fig 2H–2J) were not significantly changed by TLQP-21. Whilst energy expenditure and ambulatory activity displayed clear diurnal rhythms, there were no significant effects of TLQP-21 treatment. Similarly, UCP1 mRNA in BAT was unchanged by TLQP-21 treatment in hamsters in LP (Fig 3F).

### Expression of C3aR1 in peripheral tissues

**Fig 4:**
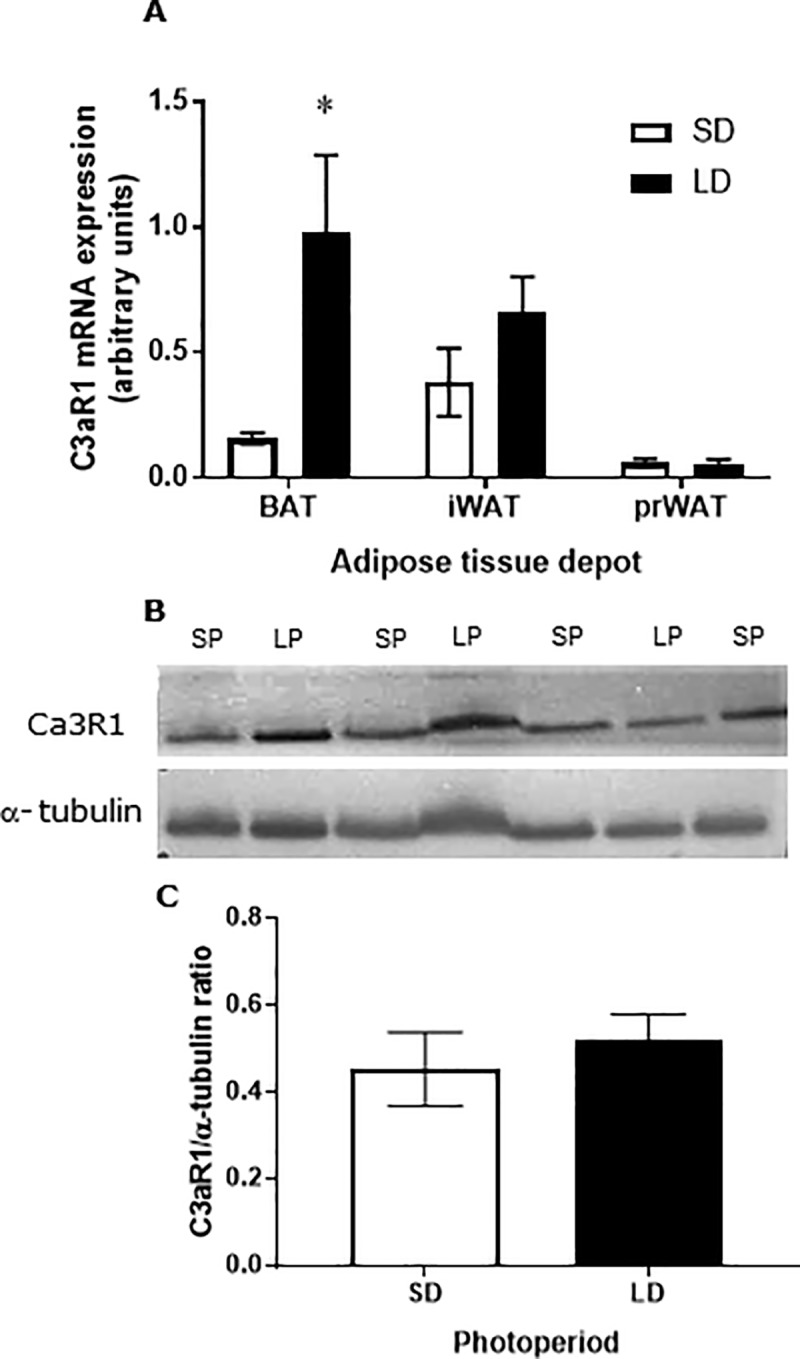
C3aR1 mRNA expression in BAT and WAT, and C3aR1 protein abundance in WAT. (**A**) Quantitative PCR analysis of C3aR1 mRNA in BAT, and interscapular (iWAT), and peri-renal (prWAT) white adipose tissue. (**B**) Representative Western blot analyses and (**C**) quantification of C3aR1 protein expression in intra-abdominal WAT from female Siberian hamsters maintained under SP or LP for 12 weeks. As reference, the 36B4 gene and α-tubulin were used to normalize mRNA and protein expression respectively. Open bars indicate SP exposure and black bars indicate LP exposure. Values are expressed as mean±SEM, n = 4/group, *P<0.05 vs LP.

We observed expression of C3aR1 mRNA in all adipose tissue depots tested. Interestingly, we observed a significant increase in mRNA levels in BAT in hamsters exposed to LP compared to SP (Fig 4A P < 0.05), but there was no difference in expression in the other WAT depots (Fig 4A). To determine whether this difference in mRNA expression translated to altered protein levels, Western blot analysis was conducted (Fig 4B and 4C). This revealed no significant photoperiodic differences in C3aR1 abundance.

### Expression of C3aR1 in the brain

**Fig 5:**
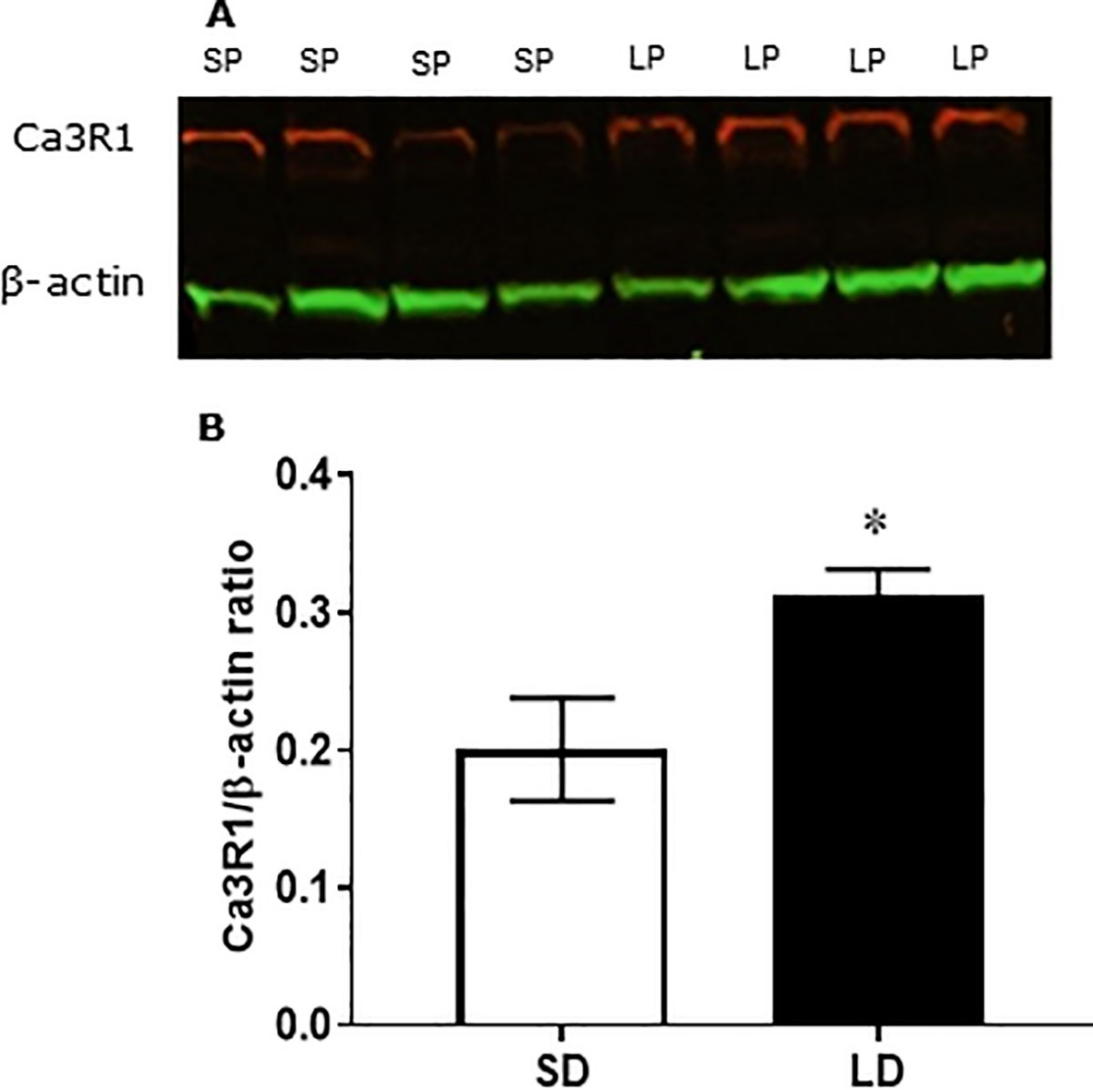
C3aR1 protein abundance in the hypothalamus. Western blot analysis (**A**) and quantification (**B**) of C3aR1 protein expression in the hypothalamus from female Siberian hamsters (n = 4/photoperiod) maintained under LP or SP for 12 weeks. Red depicts C3aR1 antibody, green depicts β-actin (normalising protein). Values are expressed as mean±SEM, *P<0.05 vs SP.

We observed comparatively high levels of expression of C3aR1 in the hypothalamus of hamsters (Fig 5A), with a 36% increase in hamsters in LP compared to those in SP (P<0.05; Fig 5B).

## Discussion

A role for TLQP-21 in the pathophysiology of gastrointestinal and metabolic disorders has been suggested by a number of independent studies [24]. Central infusion of TLQP-21 has been previously shown to induce weight loss via a reduction in food intake in Siberian hamsters in LP [8] and via increased energy expenditure in diet-induced obese (DIO) mice [9]. Interest in TLQP-21 has recently been renewed following the discovery of a cognate receptor C3aR1, which has been implicated in a number of diseases including obesity and diabetes [25]. C3aR1 mRNA expression has been observed in BAT, muscle, liver and brain, and this expression is altered by high fat feeding [25]. Therefore, to further understand the interactions between TLQP-21 and C3aR1 in the regulation of energy balance, we studied the Siberian hamster, which undergoes natural seasonal cycles of adiposity as an adaptive mechanism to survive winter [1, 19]. Body weight in hamsters exposed to SP to induce weight loss was further reduced by systemic treatment with TLQP-21, a consequence of reduced food intake together with increased energy expenditure associated with increased UCP-1 mRNA in BAT. Conversely, we observed no effects of systemic infusion of TLQP-21 in hamsters in LP, in contrast to previous observations of catabolic actions when high doses were infused centrally [8]. This lack of effect in fat hamsters in LP was surprising as studies have shown that TLQP-21 treatment decreases body weight and fat mass in DIO mice [25]. These effects have been hypothesized to occur via activation of C3aR1 and enhancement of β_3_-adrenoceptor (β_3_-AR) signalling in BAT resulting in lipid mobilization and utilization as there was no effect of systemic TLQP-21 treatment in genetically modified DIO mice lacking β_3_-AR [25]. It has been well established that SP exposure leads to an increase in β_3_-AR mRNA, which furthers augment the sympathetic drive to BAT and WAT resulting in increased sensitivity to NE, hence enhanced lipolysis [26–28]. Thus it seems likely in the current study that further enhancement of β_3_-AR via activation of C3aR1 may have occurred following TLQP-21 treatment, increasing the sympathetic drive to adipose tissue. This may possibly drive the metabolic phenotype via increased UCP1 mRNA in BAT observed in hamsters in SP treated with TLQP-21.

C3aR1 mRNA expression has been observed in WAT and liver of mice, although the expression in BAT is unknown [25, 29]. Here we observed mRNA expression of this receptor in WAT, in line with the literature, and we have now, also identified its expression in BAT in the hamster. Interestingly, the expression of C3aR1 mRNA in BAT was significantly increased in LP compared to SP, while there was no difference in its expression in WAT depots. There are undoubtedly instances where mRNA levels do not predict protein levels [30], but Western blot analyses also revealed no significant differences in C3aR1 protein abundance in WAT depots. We did observe a decrease in C3aR1 protein in the hypothalamus of hamsters exposed to SP compared to LP, which may be related to the decrease in the abundance of VGF derived peptides observed by Noli *et al* [12]. This observation suggests that the reduction in food intake and energy expenditure following systemic TLQP-21 treatment in SP may not be directly via the hypothalamus, a hypothesis supported by recent studies which reported that the hypothalamic uptake of either ^125^I-TLQP-21 or ^18^F-JMV5763 (fluorinated version of JMV5656 –a TLQP-21 analogue with sequence RRRHFHHALPPAR) was negligible [29, 31]. However, these results also suggest that the adipose tissues may not be involved in mediating the response, unless TLQP-21 is acting via alternative receptors [32], as Molteni *et al* [32] showed that JMV5656 increased in intracellular calcium in RAW264.7 macrophages was not affected by specific siRNA against C3aR1. Interestingly, recruitment of alternatively activated macrophages and eosinophils is associated with BAT activation and WAT browning via SNS activation [33].

It is also possible that the effects of systemic TLQP-21 on food intake reflect indirect actions on peripheral peptides produced by the liver, gastrointestinal tract or pancreas that that regulate appetite, rather than these being direct actions via C3aR1 in the hypothalamus. VGF mRNA has been shown to be expressed in many endocrine and neuronal cells in the gastrointestinal tract, and Turolla *et al* [31] have shown that peripheral uptake of ^18^F-JM5763 was higher in the intestine and stomach than in other tissues. Furthermore TLQP-21 dose-dependently induces contractions of gastric fundus strips but not in the distal gut portions such as the ileum or jejunum, inhibits gastric emptying and gastric acid secretion [24, 34] and has a protective effect against gastric lesioning [35]. Gastrointestinal peptides, such as CCK, GLP-1, PYY and ghrelin, are also expressed in the brain and are known to reduce food intake and increase energy expenditure [36, 37]. Interestingly all reduce gastric motility and/or acid secretion via the activation of the vagal nerve [38]. Bartolomucci *et al* [24] postulated that TLQP-21 acts in a similar manner to peripheral leptin, which acts via SNS innervation to induce reduction in gastric motility. In support, Sibilia *et al* [35] have shown that the lack of capsaicin sensitive nociceptor fibres prevents TLQP-21 from protecting the gastric mucosa against ethanol induced injury. Furthermore they showed that this effect was via nitric oxide similar to peptides such as amylin and ghrelin [35].

In conclusion, peripheral administration of TLQP-21 reduced food intake and increased energy expenditure in lean Siberian hamsters in SP, but not in fat animals exposed to LP, an effect which may reflect actions via both central and peripheral targets. An effect on central hypothalamic regulatory mechanisms might be mediated via a vagal route or via altered production of metabolic peptides, in view the apparent lack of transport of TLQP-21 across the blood-brain barrier but increased uptake in the stomach. At the same time, exogenous TLQP-21 may potentiate the effect of the endogenous adrenergic release at sympathetic nerve fibres onto adipose tissue. In view of the endogenous localization of TLQP peptides in sympathetic nerves, as well as in many endocrine locations, a combined effect of TLQP peptides released from diverse sources could operate.

## Supporting information

S1 Fig Western blots for protein expression in white adipose tissue from Siberian hamsters exposed to long and short photoperiod. (A) Ponceau staining showing the protein loading, (B) markers for western blots and (C) blot for C3aR1 and (D) blot for β-tubulin.
(JPG)

S2 Fig Western blots for protein expression in mouse inguinal adipose tissue and brown adipose tissue from Siberian hamsters exposed to long photoperiod. (A) Ponceau staining and (B) chemiluminescence blot.
(JPG)
