# Supplementary figures and images for: Photoperiodic changes in adiposity increase sensitivity of female Siberian hamsters to systemic VGF derived peptide TLQP-21
Source: PLoS One. 2019 Aug 29;14(8):e0221517. doi: 10.1371/journal.pone.0221517 (PMC6715173; doi:10.1371/journal.pone.0221517)

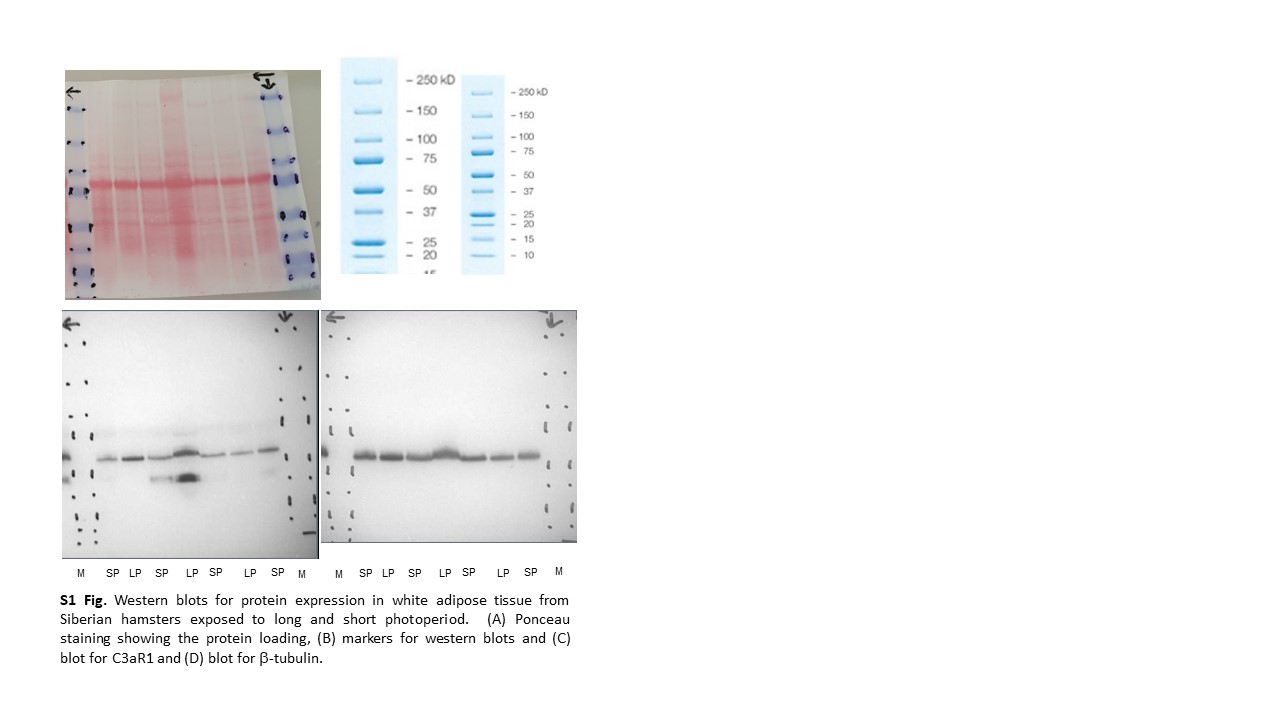

Supplement: S1 Fig — (A) Ponceau staining showing the protein loading, (B) markers for western blots and (C) blot for C3aR1 and (D) blot for β-tubulin. (JPG) [file pone.0221517.s001.jpg]

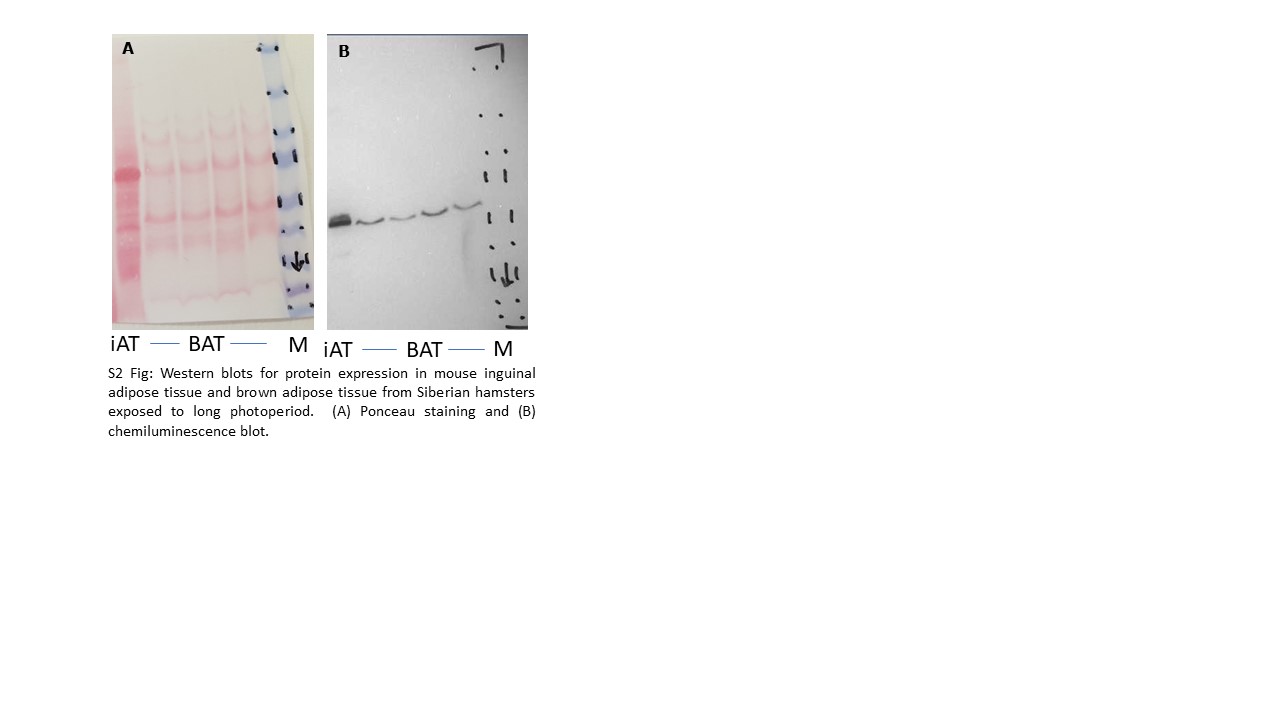

Supplement: S2 Fig — (A) Ponceau staining and (B) chemiluminescence blot. (JPG) [file pone.0221517.s002.jpg]
